# Relative Proportion Of Different Types Of Refractive Errors In Subjects Seeking Laser Vision Correction

**DOI:** 10.2174/1874364101812010053

**Published:** 2018-04-30

**Authors:** Talal A. Althomali

**Affiliations:** Associate Professor, Taif University, Taif, Saudi Arabia

**Keywords:** Relative proportion of types of refractive errors, Laser vision correction, Myopia, Hyperopia, Astigmatism, Saudi Arabia

## Abstract

**Background:**

Refractive errors are a form of optical defect affecting more than 2.3 billion people worldwide. As refractive errors are a major contributor of mild to moderate vision impairment, assessment of their relative proportion would be helpful in the strategic planning of health programs.

**Purpose:**

To determine the pattern of the relative proportion of types of refractive errors among the adult candidates seeking laser assisted refractive correction in a private clinic setting in Saudi Arabia.

**Methods:**

The clinical charts of 687 patients (1374 eyes) with mean age 27.6 ± 7.5 years who desired laser vision correction and underwent a pre-LASIK work-up were reviewed retrospectively. Refractive errors were classified as myopia, hyperopia and astigmatism. Manifest refraction spherical equivalent (MRSE) was applied to define refractive errors.

**Outcome Measures:**

Distribution percentage of different types of refractive errors; myopia, hyperopia and astigmatism.

**Results:**

The mean spherical equivalent for 1374 eyes was -3.11 ± 2.88 D. Of the total 1374 eyes, 91.8% (n = 1262) eyes had myopia, 4.7% (n = 65) eyes had hyperopia and 3.4% (n = 47) had emmetropia with astigmatism. Distribution percentage of astigmatism (cylinder error of ≥ 0.50 D) was 78.5% (1078/1374 eyes); of which % 69.1% (994/1374) had low to moderate astigmatism and 9.4% (129/1374) had high astigmatism.

**Conclusion and Relevance:**

Of the adult candidates seeking laser refractive correction in a private setting in Saudi Arabia, myopia represented greatest burden with more than 90% myopic eyes, compared to hyperopia in nearly 5% eyes. Astigmatism was present in more than 78% eyes.

## 1 INTRODUCTION

Refractive errors refer to a form of optical defect in which the optical system is unable to focus parallel rays of light sharply on retina, when the accommodation is at rest [1-3]. The most common types of refractive errors include myopia, hyperopia and astigmatism. The global magnitude of refractive errors is not reliably reported but it is estimated that more than 2.3 billion people worldwide are affected by this ocular condition [4]. Impaired vision resulting from uncorrected refractive errors is recognized as a significant health concern worldwide [5]. Refractive errors have serious social and economic impact on individuals and communities, limiting their academic and employment potential [6-9].

The pattern of refractive errors varies according to population characteristics such as, age [1, 2, 8, 10-15], gender [2, 7, 10-12, 16-18], race [19] and ethnicity [20-22]. Recent reports have suggested that the difference in the prevalence rates may be attributed to educational pressures [9, 23, 24], literacy standards [25, 26] and lifestyle changes [16] which tend to vary in urban and rural environments [3, 7, 11, 26-28]. As refractive errors are a major contributor of mild to moderate impairment of vision, assessment of their relative proportion would be helpful in strategic planning of health programs [15, 18, 29].

Currently, there is little data on the relative proportion of different types of refractive errors in Saudi Arabia. A few studies have reported the prevalence and pattern of types of refractive errors in school going children in the past decade [7, 10, 30, 31]. In the current study, we aimed at determining the pattern of relative proportion of different types of refractive errors among the adult candidates seeking laser vision correction at Tadawi Eye Surgical Centre, Taif, Saudi Arabia.

## 2 METHODS

The clinical charts of 687 patients (1374 eyes) with 335 males (670 eyes) and 352 females (704 eyes) who desired the laser vision correction and had a pre-LASIK work-up between January 2014 to June 2015 at Tadawi Eye Surgical Centre, Taif, Saudi Arabia were reviewed retrospectively. The mean age of the patients was 27.6 ± 7.5 (range 18 to 65 years): males 26.7 ± 7.5 (range; 18 to 56 years) and females 28.5 ± 7.5 (range; 18 to 68 years). Inclusion criteria were age > 18 years, no prior refractive, corneal or cataract surgery. Any cases with corneal pathologies and keratoconus were excluded from the study. The study followed the tenets of the Declaration of Helsinki and was approved by Taif University’s institutional review board with waiver of consent because the data were collected as a part of normal practice care provision.

As a part of the standard LASIK work-up, all the subjects underwent cycloplegic refraction for the measurement of refractive errors. Cycloplegic refraction was performed with 1% cyclopentolate hydrochloride. Cyclopentolate drop was instilled two times at an interval of 10 minutes, and refraction was carried out after 45 minutes from the first instillation. Cycloplegia was considered complete if the pupil was dilated to 6 mm or more and no light reflex was present. This process was followed by subjective refraction after 3 days. Manifest refraction data thus obtained were analyzed to find out the pattern of the relative distribution of different types of refractive errors.

Refractive errors were classified as myopia, hyperopia and astigmatism. Manifest refraction spherical equivalent (MRSE) was applied to define refractive errors in this study and was calculated mathematically by adding sphere power and half of the cylinder power.

Myopia was defined as a spherical equivalent of ≥ −0.50 Diopters (D) (mathematically); which was further categorized as low (≥ −0.50 D and < −3.00 D), moderate (≥ −3.00 D and < −6.00 D) and high (≥ −6.00 D). Hyperopia was defined as a spherical equivalent of ≥ +0.50 D; which was further categorized as low to moderate (≥ +0.50 D and < +3.00 D) and high (≥ +3.0 D) hyperopia. Emmetropia with astigmatism was defined as absolute cylindrical error of ≥ 0.50 diopter cylinder (DC) but had emmetropia when spherical equivalent was considered (MRSE; > −0.5 D to < +0.5 D).

Astigmatism was defined as cylinder error of ≥ 0.50 DC (absolute value) in any axis. Low to moderate astigmatism was defined as cylinder error of ≥ 0.50 DC and < 3.00 DC and high astigmatism as ≥ 3.00 DC. Distribution of astigmatism was also analyzed on the basis of axis of the principal meridians. Astigmatism was classified as With The Rule (WTR) if the axis of positive cylinder lied within 30 degrees (°) on either side of vertical meridian, Against The Rule (ATR) if the axis of positive cylinder lied within 30° on either side of horizontal meridian and oblique if the axis lied between 120° to 150° and 30° to 60°.

Based on the focus of the principal meridians, the astigmatism was classified into simple (myopic/hyperopic), compound (myopic/hyperopic) and mixed astigmatism. Simple myopic astigmatism was defined as plano sphere (< −0.5 D to < +0.5 D) and cylinder of ≥ −0.50 DC, simple hyperopic astigmatism was defined as plano sphere (< −0.5 D to < +0.5 D) and cylinder of ≥ +0.50 DC); compound myopic astigmatism was defined as sphere of ≥ −0.5 D and cylinder of ≥ −0.50 D, compound hyperopic astigmatism was defined as sphere of ≥ +0.5 D and cylinder of ≥ +0.50 DC. Astigmatism was defined as mixed if the sphere was positive (> +0.5 D) and cylinder value was negative (> −0.50 D) or vice versa and the cylinder value was greater than a sphere. Data were analyzed with Microsoft Excel 2013 (Microsoft, Redmond, WA).

## 3 RESULTS

The mean spherical equivalent for 1374 eyes was −3.11 ± 2.88 D (males = −2.72 ± 2.63 D, females = −3.47 ± 3.06 D). Of the total 1374 eyes, 91.8% (n = 1262) eyes had myopia, 4.7% (n = 65) eyes had hyperopia and 3.4% (n = 47) had emmetropia with astigmatism. Distribution of myopia, hyperopia and emmetropia with astigmatism is presented for different populations *i.e.* overall, male/female and age up to/above 40 years. (Table **1**).

Overall distribution of astigmatism (cylinder error of ≥ 0.50 D) in the current study population was found to be 78.5% (1078/1374 eyes). Distribution of different types of astigmatism categories is presented in detail in Table (**2**) for different populations (overall, male/female and age up to/above 40 years).

## 4 DISCUSSION

The prevalence and proportion of different types of refractive errors vary according to different ethnical, cultural, geographical, demographic, ocular, economical [23], and environmental (education [26], prolonged indoor and near activities [27]) characteristics among various studies [3, 15, 32]. Before comparing the current study results with other studies, it is important to acknowledge differences in the definitions, study population, methodologies and refractive error measurement techniques. While the majority of previous publications studying refractive errors have been performed in school children and a few in general population (comprising different age groups), the current study population included those adults who already had at least some refractive error and desired laser refractive correction. Therefore, the relative proportions of different types of refractive errors, as found in the current study and in the literature are being discussed here. Of note, the prevalence of different types of refractive errors in the representative age group or in general population have not been studied/discussed. A review of literature reveals different minimum/maximum cut-off values to define the refractive errors [7, 10, 17, 21, 32, 33] and data analysis methods (patient wise versus eye wise distribution) [7, 13, 30, 34, 35] *etc*. These differences will be highlighted while comparing the current study results with the literature.

In the current study, we determined the proportion of different types of refractive errors in the patients who desired laser vision correction. The results of the current study indicate that the myopia accounts for 91.85% eyes, hyperopia in 4.73% eyes and emmetropia with astigmatism only in 3.42% eyes of patients who had a pre-LASIK work up. Of the total eyes, 78.46% had astigmatism and 21.54% eyes had no astigmatism.

A few publications have reported the proportion of types of refractive errors in their study population. Three studies from Saudi Arabia [7, 30, 31] have reported the proportion of myopia ranging from 55 to 65.7%, hyperopia ranging from 9.9 to 45% and astigmatism ranging from 25 to 66.20% Table (**3**). The pattern of distribution of refractive errors found in the current study is similar to these studies; however, the overall proportion of myopia and astigmatism is comparatively lower and hyperopia is higher in these studies than the current one. Although there is a geographical similarity between the current study and the studies mentioned above, the comparative analysis with the current study is inappropriate, most likely due to the differences in the age group of study population (school children/adolescent versus patients seeking laser vision correction).

The proportion of distribution of refractive error is also available from other parts of the world, such as countries like Nigeria [2, 34], Ghana [13], Uganda [36], Nepal [28] and Pakistan [16] Table (**3**). The results from these studies couldn’t be adduced in comparison to the current study due to the inherent differences in the methodological characteristics of each study Table (**3**). Most of the authors have considered the refractive error of only one eye (worse eye or right/left eye), whereas some authors have not described how the pattern of refractive errors was calculated when both eyes of a patient were considered. In a study involving both the eyes, mean MRSE of both eyes of one subject was considered for analyzing the pattern of types of refractive error [35], which the author feels is inappropriate. It is recommended that the types of refractive error should be decided eye wise instead of taking mean MRSE of right and left eye. In the current study, the refractive error data has been analyzed eye wise instead of patient wise.

There is lack of agreement among different studies regarding the definition of myopia and hyperopia. While most of the authors have considered MRSE as the defining criterion [1, 11, 12, 17, 21, 24-26, 29, 37], others have included the spherical errors only [2, 13, 38]. Such a method of presentation may potentially result in the under-representation of myopic/hyperopic refractive errors in a population as only the patients with no astigmatism (< 0.5 D) will be categorized as myopia/hyperopia. This method will not include the eyes with simple/compound myopic/hyperopic astigmatism under the proportion of myopia/hyperopia. Thus, care should be taken when comparing the results of such studies.

There is lack of uniformity among the available publications regarding the lower and upper cut-off point taken for diagnosing the type of refractive error. For myopia, various lower cut-off values of MRSE have been used in the literature, such as > −0.50 D [11, 14, 21, 24], ≥□−0.50 D [1, 8, 10, 12, 17, 27, 39], ≥ −0.75 D [7] and ≥ −1.00 D [33]. The corresponding values for hyperopia have been reported as > +0.50 D [11, 14, 21, 24, 30], ≥ +0.5 D [1, 12, 17, 26], ≥ +1.50 D [23], ≥ +1.75 D [33] and ≥ +2.0 D [7, 8, 10, 18, 27, 39]. Likewise, for astigmatism, minimum cylinder (positive cylinder format) cut-off values of > 0.5 DC [8, 11, 21, 24, 25, 29], ≥ 0.5 DC [17, 40], ≥ 0.75 D [10, 12, 18, 26, 39], ≥ 1.00 DC [33] and ≥ 1.5 DC [22, 32] have been used in different studies. In addition, various ranges of refractive error have been used for further sub-classification of myopia, hyperopia and astigmatism. For example, for low myopia category, some authors have used MRSE range of −0.5 D to < −3.0 D [10, 12, 30], whereas others have used ≥ −0.5 to −2.75 D (sphere) [2]. In the current study, we have used definitions of refractive errors as recommended by American Academy of Ophthalmology with a few modifications [41]. As such, there is a need to standardize the definitions for diagnosing refractive errors.

A review of the literature reveals that some papers do not provide detailed definitions for different astigmatism categories: simple myopic/hyperopic, compound myopic/hyperopic, mixed astigmatism [2, 34, 42, 43] which may cause some errors in calculations. Few studies stated that for mixed astigmatism, sphere should be positive (>0.5 D) and cylinder value should be negative (>−0.50 D) or vice versa [2, 34]. If we consider this definition, a patient with sphere value of +2 D and cylinder value of −1 D, will get classified as mixed astigmatism, despite the fact that the patient actually has compound hyperopic astigmatism. This can be understood by transposing the refractive error to the reduced state (*i.e.* with lower absolute sphere value). After transposition, both sphere and cylinder values become +1 D, which means the patient actually has compound hyperopic astigmatism. Similarly, a patient with −2 D sphere and +1 D cylinder has compound myopic astigmatism and not the mixed. Another error can happen if magnitudes of sphere and cylinder values are same with different signs, *e.g.* −2 D sphere with +2 D cylinder or +2 D sphere with −2 D cylinder. Again, these cases seem to have the mixed astigmatism, which is not true. After transposition to the reduced state, the sphere and cylinder values will respectively become 0 D and +1 D for the first case and 0 D and −1 D for the second case. Thus, these subjects actually have simple hyperopic and simple myopic astigmatism respectively. Alternatively, for classification as mixed astigmatism, the cylinder value must be greater than sphere; otherwise, astigmatism will be compound/simple myopic/hyperopic depending upon the sign and magnitude of sphere and cylinder.

From the results of the current study, it is evident that the majority of people with some refractive error, desiring laser vision correction are myopic. More than 40% eyes had moderate to high myopia. In fact, these eyes pose high risk for ectasia development post refractive surgery; patients should be duly informed about these complications. Other alternative methods of refractive correction, such as phakic intraocular lens implantation can be considered in these eyes to avoid ablation related complications. Additionally, in the current study, more than 9% eyes had high astigmatism of ≥ 3 D. While it is crucial to account for cyclotorsion correction in all astigmatic eyes when performing laser refractive surgery, it is of utmost importance in eyes with high astigmatism.

## CONCLUSION

This study estimates the proportion of types of refractive error among individuals with at least some refractive error who visited laser surgical center desiring laser vision correction. At 90%, myopia represented the greatest burden, compared to hyperopia in nearly 5% eyes. Astigmatism was prevalent in more than 78% eyes. The choice of treatment to achieve spectacle independence should be based on patient’s individual needs and risk profiles. There is a need to standardize the definitions used for categorizing refractive errors so as to facilitate comparison between different studies.

## Figures and Tables

**Table 1:** Proportion of types of refractive errors (Myopia, Hyperopia and Emmetropia with astigmatism).

**Types of refractive errors**		**Distribution**																			
		**Overall (N=1374)** ****(All percentages under this column calculated with N = 1374)				**Gender Based Distribution**								**Age based Distribution**							
						**Males (N =670)** ****(All percentages under this column calculated with N = 670)				**Females (N = 704)** ****(All percentages under this column calculated with N = 704)				**Age ≤ 40 (N =1284)** ****(All percentages under this column calculated with N = 1284)				**Age >40 (N = 90)** ****(All percentages under this column calculated with N = 90)			
		**N**	**%**	**N**	**%**	**N**	**%**	**N**	**%**	**N**	**%**	**N**	**%**	**N**	**%**	**N**	**%**	**N**	**%**	**N**	**%**
**Myopia** **(MRSE ** **≤ -0.50 D)**	Low myopia (≤ −0.50 D and > −3.00 D)	1262	91.8%	702	51.1%	609	90.9%	390	58.2%	653	92.8%	312	44.3%	1195	93.1%	620	48.3%	67	74.4%	41	45.6%
	Moderate myopia (≤ −3.00 D and > −6.00 D)			400	29.1%			158	23.6%			242	34.4%			414	32.2%			16	17.8%
	High myopia (≤ −6.00 D)			160	11.6%			61	9.1%			99	14.1%			161	12.5%			10	11.1%
**Hyperopia** **(MRSE ** **≥ +0.50 D)**	Low to moderate hyperopia (≥ +0.50 D and < +3.00 D)	65	4.7%	45	3.3%	33	4.9%	25	3.7%	32	4.5%	20	2.8%	46	3.6%	29	2.3%	19	21.1%	16	17.8%
	High hyperopia (≥ +3.0 D)			20	1.5%			8	1.2%			12	1.7%			17	1.3%			3	3.3%
**Emmetropia with astigmatism**	(MRSE; > −0.5 D to < +0.5 D), but cylinder ≥ 0.50 DC)	47	3.4%			28	4.18%			19	2.7%			43	3.35%			4	4.4%		
MRSE- Manifest refraction spherical equivalent, D- Diopter, DC- Diopter cylinder																					

**Table 2:** Proportion of different categories of astigmatism.

**Types of refractive errors**		**Distribution**																			
		**Overall (N=1374)** ****(All percentages under this column calculated with N = 1374)				**Gender Based Distribution**								**Age based Distribution**							
						**Males (N =670)** ****(All percentages under this column calculated with N = 670)				**Females (N = 704)** ****(All percentages under this column calculated with N = 704)				**Age ≤ 40 (N =1284)** ****(All percentages under this column calculated with N = 1284)				**Age >40 (N = 90)** ****(All percentages under this column calculated with N = 90)			
		**N**	**%**	**N**	**%**	**N**	**%**	**N**	**%**	**N**	**%**	**N**	**%**	**N**	**%**	**N**	**%**	**N**	**%**	**N**	**%**
**Astigmatism** **(Cylinder ≥ 0.50 DC)**	Low to moderate Astigmatism^*^ (≥ 0.50 DC and < 3.00 DC)	1078	78.5%	949	69.1%	534	79.7%	472	77.5%	544	77.3%	477	73.0%	1017	79.2%	897	69.9%	61	67.8%	52	57.8%
	High Astigmatism^*^ (≥ 3.00 DC)			129	9.4%			62	10.2%			67	10.3%			120	9.3%			9	10.0%
	WTR^*^ (+/- 30° on 90°; cylinder ≥ 0.50 DC)			784	57.1%			354	58.1%			430	65.8%			748	58.3%			36	40.0%
	ATR^*^ (+/- 30° on 180°); cylinder ≥ 0.50 DC)			182	13.2%			117	19.2%			65	10.0%			168	13.1%			14	15.6%
	OBL^*^ (120° to 150° and 30° to 60; cylinder ≥ 0.50 DC)			112	8.2%			63	10.3%			49	7.5%			101	7.9%			11	12.2%
	Simple Myopic [plano sphere (> −0.5 D to < +0.5 D) & Cyl (-ve) ≤ −0.5 DC]			112	8.2%			73	12.0%			39	6.0%			105	8.2%			7	7.8%
	Simple Hyperopic [plano sphere (> -0.5 D to < +0.5 D) & Cyl (+ve) ≥ 0.5 DC]			17	1.2%			12	2.0%			5	0.8%			12	0.9%			5	5.6%
	Compound Myopic (sph ≤ −0.5 D & cyl ≤ −0.50 DC)			895	65.1%			419	68.8%			476	72.9%			850	66.2%			45	50.0%
	Compound Hyperopic (sphere of ≥ +0.5 D & cylinder of ≥ +0.50 DC)			29	2.1%			14	2.3%			15	2.3%			27	2.1%			2	2.2%
	Mixed astigmatism^^^			25	1.8%			16	2.6%			9	1.4%			23	1.8%			2	2.2%
**No Astigmatism**	(Absolute cylinder ≤ 0.50 DC)	296	21.5%	-	-	136	20.3%	-	-	160	22.7%	-	-	267	20.8%	-	-	29	32.2%	-	-

WTR- With the rule ATR- Against the rule, OBL- Oblique, D- Diopter, DC- Diopter cylinder, ^ *^Absolute values, ^^^Astigmatism is mixed if sphere is positive (> 0.5 D) and cylinder value is negative (< -0.50 D) or vice versa and the cylinder > sphere, Cyl= cylinder, - Not applicable

**Table 3:** Peer reviewed studies presenting the proportion of types of refractive errors.

**Author (year)** **(Study population;** **Set up)**	**Age group** **(Y)**	**Sample size (Eyes)**	**Definition Criteria for myopia/ hyperopia**	**Definition/cut-off for Refractive errors**	**Proportion**		
					**Myopia**	**Hyperopia**	**Astigmatism**
**^†^Rowaily (2010)** **(Adolescents;** **Riyadh, Saudi Arabia)^31^**	12-13	1536 M=734 F=802 **one or both eyes**	SEQ	**Myopia:** Mild (-0.5 to <-3D); Mod(-3 to <-6D); High (>-6D) **hyperopia:** Mild (+0.5 to <+3D); Mod (+3 to <+6D); High (>+6D)	Overall=57.6% Mild=45.70% Mod=6.60% High=3.30%	15.2% Mild=9.90% Mod=64.60% High=0.70%	66.20%
**Al Wadaani (2013)** **(Primary school children;** Saudi Arabia) [7]	6-14	2002 **one or both eyes**	SEQ	**Myopia:** ≤-0.75D **Hyperopia: ≥**+2D **Astig:** ≥0.50DC or ≥1.00DC	65.70%	9.90%	Overall=24.45% MA=12.4% HA=12.1%
**Al Rowaily (2010)** **(Pre-school children;** **Riyadh, Saudi Arabia) [****30****]**	4-8	1319 M=577 F=742 **one or both eyes**	SEQ	**Myopia:** Mild (-0.5 to <-3D); Mod (-3 to <-6D); High (>-6D) **hyperopia:** Mild (+0.5 to <+3D); Mod (+3 to <+6D); High (>+6D)	Overall=55% Mild=75.80% Mod=12.10% High=12.10%	Overall=45% Mild=85.20% Mod=11.10% High=3.70%	55%
**Emmanuel (2013)** **(School children;** **Nigeria) [****34****]**	9-21	1175 (2350) F= 54.5% **both eyes**	Sphere	**Astig:** >0.50DC	29.50%	13.10%	Overall=57.4% WTR=71.4% ATR=22.9% OBL=5.7%
**Ovenseri-Ogbomo (2010)** **(School children;** **Ghana [****13****]**	5-19	957 M= 31% F= 69% **right eyes**	Sphere	**Hyperopia**= ≥+2.00 DS **myopia**= ≤−0.50 DS **astigmatism**= ≤−0.50 DC	27.00%	18.00%	55.00%
**Opubiri (2013)** **(Tertiary hospital;** **South Nigeria) [****2****]**	4-15	506 114 had RE **laterality not mentioned**	Sphere	**Myopia** Low (≥-0.5DS to -2.75DS), Mod (–3.0DS to –5.0DS), High (>-5.0 DS) **Hyperopia** Low (≥+0.5DS) to +2.75DS), Mod (+3.0DS to +5.0DS), High (>+5.0DS) **Astig (-ve)** (≥0.5DC in any axis) SMA (PlanoDS/≥-0.50DC); CMA (≥-0.5DS/≥-0.5DC); MIX: (≥+0.5DS/≥-0.5DC)	61.40%	11.40%	Overall=27.2% SMA=10.5% CMA=12.3% MIX=4.4%
**Pokharel (2010)** **(School children; Nepal) [****28****]**	7-15	440 **urban=220** (107 M, 113 F) **rural= 220** (101 M, 119 F) **both eyes**	not clearly defined	**Myopia:** Low (>-0.50 to <-3.0D); Mod (>-3.0 to <-6.0D); High(>-6.0D) **hyperopia:** Mild (+0.5 to <+3D); Mod. (>+3 to <+6D); High (>+6D) **Astigmatism:** any cylindrical error	59.80%	31.00%	9.20%
**Kawuma (2002)** **(Primary school children;** **Uganda) [****36****]**	6-9	623 M= 301 F= 322 **one or both eyes**	not clearly defined	refractive error of ±0.50 or worse in one or both eyes	11%	37%	52%
**Ali (2007)** **(School children;** **Lahore, Pakistan) [****16****]**	11-16	540 **one or both eyes**	not provided	_	43%	21.50%	Overall=35.5% SA=21.50% CA+Mix=14%

^†^27.20% subjects had emmetropia, MA= Myopic Astigmatism; LVC= Laser Vision Correction; M= Male; F= Female; SEQ= Spherical Equivalent; DC= Diopter cylinder; DS= Diopter sphere; Astig= Astigmatism ; Y= years; RE= Refractive error; HA= Hyperopic Astigmatism; SA= Simple Astigmatism; CA= Compound Astigmatism; SMA= Simple Myopic Astigmatism; CMA= Compound Myopic Astigmatism; WTR= With the rule; ATR= Against the rule; OBL= Oblique; Mod= Moderate

## References

[1] Jobke S., Kasten E., Vorwerk C. (2008). The prevalence rates of refractive errors among children, adolescents, and adults in Germany.. Clin. Ophthalmol..

[2] Opubiri I., Adio A., Megbelayin E. (2013). Refractive error pattern of children in South-South Nigeria: A tertiary hospital study.. Sky J Med & Med Sci..

[3] Rai S., Thapa H.B., Sharma M.K., Dhakhwa K., Karki R. (2012). The distribution of refractive errors among children attending Lumbini Eye Institute, Nepal.. Nepal. J. Ophthalmol..

[4] Holden BA, Sulaiman S, Knox K (2000). The challenge of providing spectacles in the developing world. Community eye health / International Centre for Eye Health..

[5] Resnikoff S., Pascolini D., Mariotti S.P., Pokharel G.P. (2008). Global magnitude of visual impairment caused by uncorrected refractive errors in 2004.. Bull. World Health Organ..

[6] Abbasi S., Imtiaz A., Shah A.R., Zamir Q. (2013). Frequency of amount and axis of astigmatism in subjects of Rawalpindi, Pakistan.. J. Pak. Med. Assoc..

[7] Al Wadaani F.A., Amin T.T., Ali A., Khan A.R. (2012). Prevalence and pattern of refractive errors among primary school children in Al Hassa, Saudi Arabia.. Glob. J. Health Sci..

[8] Mehari Z.A., Yimer A.W. (2013). Prevalence of refractive errors among schoolchildren in rural central Ethiopia.. Clin. Exp. Optom..

[9] Shrestha G.S., Sujakhu D., Joshi P. (2011). Refractive error among school children in Jhapa, Nepal.. J. Optom..

[10] Aldebasi Y.H. (2014). Prevalence of correctable visual impairment in primary school children in Qassim Province, Saudi Arabia.. J. Optom..

[11] Krishnaiah S., Srinivas M., Khanna R.C., Rao G.N. (2009). Prevalence and risk factors for refractive errors in the South Indian adult population: The Andhra Pradesh Eye disease study.. Clin. Ophthalmol..

[12] Ostadimoghaddam H., Fotouhi A., Hashemi H., Yekta A., Heravian J., Rezvan F., Ghadimi H., Rezvan B., Khabazkhoob M. (2011). Prevalence of the refractive errors by age and gender: The Mashhad eye study of Iran.. Clin. Experiment. Ophthalmol..

[13] Ovenseri-Ogbomo G., Omuemu V. (2010). Prevalence of refractive error among school children in the Cape Coast Municipality, Ghana.. Clin Optom..

[14] Rani P.K., Raman R., Rachapalli S.R., Kulothungan V., Kumaramanickavel G., Sharma T. (2010). Prevalence of refractive errors and associated risk factors in subjects with type 2 diabetes mellitus SN-DREAMS, report 18.. Ophthalmology.

[15] Shrestha S.P., Bhat K.S., Binu V.S., Barthakur R., Natarajan M., Subba S.H. (2010). Pattern of refractive errors among the Nepalese population: A retrospective study.. Nepal. J. Ophthalmol..

[16] Ali A., Ahmed I., Ayub S. (2007). Prevalence of undetected refractive errors among school children.. Biomedica.

[17] Ferraz FH, Corrente JE, Opromolla P, Padovani CR, Schellini SA (2015). Refractive errors in a Brazilian population: Age and sex distribution.. Ophthalmic & physiological optics: J British Coll Oph Opti..

[18] Yekta A., Fotouhi A., Hashemi H., Dehghani C., Ostadimoghaddam H., Heravian J., Derakhshan A., Yekta R., Behnia M., Khabazkhoob M. (2010). Prevalence of refractive errors among schoolchildren in Shiraz, Iran.. Clin. Experiment. Ophthalmol..

[19] Qiu M., Wang S.Y., Singh K., Lin S.C. (2014). Racial disparities in uncorrected and undercorrected refractive error in the United States.. Invest. Ophthalmol. Vis. Sci..

[20] Fozailoff A., Tarczy-Hornoch K., Cotter S., Wen G., Lin J., Borchert M., Azen S., Varma R., Writing Committee for the MEPEDS Study Group (2011). Prevalence of astigmatism in 6- to 72-month-old African American and Hispanic children: The Multi-ethnic Pediatric Eye Disease Study.. Ophthalmology.

[21] Pan C.W., Zheng Y.F., Anuar A.R., Chew M., Gazzard G., Aung T., Cheng C.Y., Wong T.Y., Saw S.M. (2013). Prevalence of refractive errors in a multiethnic Asian population: the Singapore epidemiology of eye disease study.. Invest. Ophthalmol. Vis. Sci..

[22] Wen G., Tarczy-Hornoch K., McKean-Cowdin R., Cotter S.A., Borchert M., Lin J., Kim J., Varma R., Multi-Ethnic Pediatric Eye Disease Study Group (2013). Prevalence of myopia, hyperopia, and astigmatism in non-Hispanic white and Asian children: Multi-ethnic pediatric eye disease study.. Ophthalmology.

[23] Pi L.H., Chen L., Liu Q., Ke N., Fang J., Zhang S., Xiao J., Ye W.J., Xiong Y., Shi H., Yin Z.Q. (2010). Refractive status and prevalence of refractive errors in suburban school-age children.. Int. J. Med. Sci..

[24] Ziaei H., Katibeh M., Solaimanizad R., Hosseini S., Gilasi H.R., Golbafian F., Javadi M.A. (2013). Prevalence of refractive errors; the yazd eye study.. J. Ophthalmic Vis. Res..

[25] Cheng C.Y., Hsu W.M., Liu J.H., Tsai S.Y., Chou P. (2003). Refractive errors in an elderly Chinese population in Taiwan: The Shihpai Eye Study.. Invest. Ophthalmol. Vis. Sci..

[26] Ezelum C., Razavi H., Sivasubramaniam S., Gilbert C.E., Murthy G.V., Entekume G., Abubakar T., Nigeria National Blindness and Visual Impairment Study Group (2011). Refractive error in Nigerian adults: Prevalence, type, and spectacle coverage.. Invest. Ophthalmol. Vis. Sci..

[27] Paudel P., Ramson P., Naduvilath T., Wilson D., Phuong H.T., Ho S.M., Giap N.V. (2014). Prevalence of vision impairment and refractive error in school children in Ba Ria - Vung Tau province, Vietnam.. Clin. Experiment. Ophthalmol..

[28] Pokharel A., Pokharel P.K., Das H., Adhikari S. (2010). The patterns of refractive errors among the school children of rural and urban settings in Nepal.. Nepal. J. Ophthalmol..

[29] Raju P., Ramesh S.V., Arvind H., George R., Baskaran M., Paul P.G., Kumaramanickavel G., McCarty C., Vijaya L. (2004). Prevalence of refractive errors in a rural South Indian population.. Invest. Ophthalmol. Vis. Sci..

[30] Al-Rowaily MA (2010). Prevalence of refractive errors among pre-school children at King Abdulaziz Medical City, Riyadh, Saudi Arabia.. Saudi journal of ophthalmology : official journal of the Saudi Ophthalmological Society..

[31] Rowaily MAA, Alanizi BM (2012). Prevalence of uncorrected refractive errors among adolescents at king abdul-aziz medical city, riyadh, saudi arabia.. Journal of Clinical & Experimental Ophthalmology..

[32] Dirani M., Chan Y.H., Gazzard G., Hornbeak D.M., Leo S.W., Selvaraj P., Zhou B., Young T.L., Mitchell P., Varma R., Wong T.Y., Saw S.M. (2010). Prevalence of refractive error in Singaporean Chinese children: The strabismus, amblyopia, and refractive error in young Singaporean Children (STARS) study.. Invest. Ophthalmol. Vis. Sci..

[33] Wang X., Liu D., Feng R., Zhao H., Wang Q. (2014). Refractive error among urban preschool children in Xuzhou, China.. Int. J. Clin. Exp. Pathol..

[34] Emmanuel M.O., Dennis N.G., Anya K. (2013). Pattern of refractive astigmatism in Nigerian high schools.. Sky Journal of Medicine and Medical Sciences..

[35] Williams K.M., Verhoeven V.J., Cumberland P., Bertelsen G., Wolfram C., Buitendijk G.H., Hofman A., van Duijn C.M., Vingerling J.R., Kuijpers R.W., Höhn R., Mirshahi A., Khawaja A.P., Luben R.N., Erke M.G., von Hanno T., Mahroo O., Hogg R., Gieger C., Cougnard-Grégoire A., Anastasopoulos E., Bron A., Dartigues J.F., Korobelnik J.F., Creuzot-Garcher C., Topouzis F., Delcourt C., Rahi J., Meitinger T., Fletcher A., Foster P.J., Pfeiffer N., Klaver C.C., Hammond C.J. (2015). Prevalence of refractive error in Europe: The European Eye Epidemiology (E(3)) Consortium.. Eur. J. Epidemiol..

[36] Kawuma M., Mayeku R. (2002). A survey of the prevalence of refractive errors among children in lower primary schools in Kampala district.. Afr. Health Sci..

[37] Wu S.Y., Nemesure B., Leske M.C. (1999). Refractive errors in a black adult population: The Barbados Eye Study.. Invest. Ophthalmol. Vis. Sci..

[38] Cruz-Becerril A., Valdivia A., Peralta R., Castro-Reyes M.A. (2015). Prevalence of refractive errors in Mexican patients with keratoconus.. Clinical Optometry..

[39] Maul E., Barroso S., Munoz S.R., Sperduto R.D., Ellwein L.B. (2000). Refractive Error Study in Children: Results from La Florida, Chile.. Am. J. Ophthalmol..

[40] Tong L, Saw SM, Carkeet A, Chan WY, Wu HM, Tan D (2002). Prevalence rates and epidemiological risk factors for astigmatism in Singapore school children..

[41] (2013). American. American Academy of Ophthalmology Refractive Management/Intervention Panel. Preferred Practice Pattern®Guidelines.. Refractive Errors & Refractive Surgery (2013): American Academy of Ophthalmology, San Francisco, CA. Available at: www.aao.org/ppp.

[42] Shih YF, Hsiao CK, Tung YL, Lin LL, Chen CJ, Hung PT (2004). The prevalence of astigmatism in Taiwan schoolchildren.. Optometry and vision science :Official publication of the American Academy of Optometry..

[43] Ansari  MZ-u-H, Ali  A., Afaq A., Ahmad T., Sharif-ul-Hasan K. (2009). Relative distribution and amount of different types of astigmatism in mixed ethnic population of karachi.. Pak J. Ophthalmol..

